# Supplementary material for: MetaQTL: a package of new computational methods for the meta-analysis of QTL mapping experiments
Source: BMC Bioinformatics. 2007 Feb 8;8:49. doi: 10.1186/1471-2105-8-49 (PMC1808479; doi:10.1186/1471-2105-8-49)
Supplement: Additional File 7 — MetaQTL Package : jar file and tutorial. This Zip archive contains both the MetaQTL JAR file and the files of the tutorial. [file 1471-2105-8-49-S7.zip › org.inra.metaqtl/doc/org/metaqtl/main/CmdLineParser.html]

CmdLineParser


|  |  |  |
| --- | --- | --- |
| **Overview** \| **Package** \| **Class** \| **Use** \| **Tree** \| **Deprecated** \| **Index** \| **Help** | |  |
| **PREV CLASS**   **NEXT CLASS** | **FRAMES**    **NO FRAMES**     **All Classes** |  |
| SUMMARY: NESTED \| FIELD \| CONSTR \| METHOD | DETAIL: FIELD \| CONSTR \| METHOD |  |


---


## org.metaqtl.main Class CmdLineParser

```
java.lang.Object
  org.metaqtl.main.CmdLineParser
```

---

``` public class CmdLineParser extends java.lang.Object ```

Largely GNU-compatible command-line options parser. Has short (-v) and
long-form (--verbose) option support, and also allows options with
associated values (-d 2, --debug 2, --debug=2). Option processing
can be explicitly terminated by the argument '--'.

**Version:**
:   $Revision: 1.2 $

**Author:**
:   Steve Purcell

**See Also:**: `jargs.examples.gnu.OptionTest`

---

| **Nested Class Summary** | |
| --- | --- |
| `static class` | `CmdLineParser.IllegalOptionValueException`             Thrown when an illegal or missing value is given by the user for an option that takes a value. |
| `static class` | `CmdLineParser.NotFlagException`             Thrown when the parsed commandline contains multiple concatenated short options, such as -abcd, where one or more requires a value. |
| `static class` | `CmdLineParser.Option`             Representation of a command-line option |
| `static class` | `CmdLineParser.OptionException`             Base class for exceptions that may be thrown when options are parsed |
| `static class` | `CmdLineParser.UnknownOptionException`             Thrown when the parsed command-line contains an option that is not recognised. |
| `static class` | `CmdLineParser.UnknownSuboptionException`             Thrown when the parsed commandline contains multiple concatenated short options, such as -abcd, where one is unknown. |


**Constructor Summary**

`CmdLineParser()`


| **Method Summary** | |
| --- | --- |
| `CmdLineParser.Option` | `addBooleanOption(char shortForm, java.lang.String longForm)`             Convenience method for adding a boolean option. |
| `CmdLineParser.Option` | `addBooleanOption(java.lang.String longForm)`             Convenience method for adding a boolean option. |
| `CmdLineParser.Option` | `addDoubleOption(char shortForm, java.lang.String longForm)`             Convenience method for adding a double option. |
| `CmdLineParser.Option` | `addDoubleOption(java.lang.String longForm)`             Convenience method for adding a double option. |
| `CmdLineParser.Option` | `addIntegerOption(char shortForm, java.lang.String longForm)`             Convenience method for adding an integer option. |
| `CmdLineParser.Option` | `addIntegerOption(java.lang.String longForm)`             Convenience method for adding an integer option. |
| `CmdLineParser.Option` | `addLongOption(char shortForm, java.lang.String longForm)`             Convenience method for adding a long integer option. |
| `CmdLineParser.Option` | `addLongOption(java.lang.String longForm)`             Convenience method for adding a long integer option. |
| `CmdLineParser.Option` | `addOption(CmdLineParser.Option opt)`             Add the specified Option to the list of accepted options |
| `CmdLineParser.Option` | `addStringOption(char shortForm, java.lang.String longForm)`             Convenience method for adding a string option. |
| `CmdLineParser.Option` | `addStringOption(java.lang.String longForm)`             Convenience method for adding a string option. |
| `java.lang.Object` | `getOptionValue(CmdLineParser.Option o)`             Equivalent to `getOptionValue(o, null)`. |
| `java.lang.Object` | `getOptionValue(CmdLineParser.Option o, java.lang.Object def)` |
| `java.util.Vector` | `getOptionValues(CmdLineParser.Option option)` |
| `java.lang.String[]` | `getRemainingArgs()` |
| `void` | `parse(java.lang.String[] argv)`             Extract the options and non-option arguments from the given list of command-line arguments. |
| `void` | `parse(java.lang.String[] argv, java.util.Locale locale)`             Extract the options and non-option arguments from the given list of command-line arguments. |

**Methods inherited from class java.lang.Object**

`clone, equals, finalize, getClass, hashCode, notify, notifyAll, toString, wait, wait, wait`

**Constructor Detail**

### CmdLineParser

```
public CmdLineParser()
```


**Method Detail**

### addOption

```
public final CmdLineParser.Option addOption(CmdLineParser.Option opt)
```

:   Add the specified Option to the list of accepted options

---


### addStringOption

```
public final CmdLineParser.Option addStringOption(char shortForm,
                                                  java.lang.String longForm)
```

:   Convenience method for adding a string option.

    :   **Returns:**: the new Option

---


### addStringOption

```
public final CmdLineParser.Option addStringOption(java.lang.String longForm)
```

:   Convenience method for adding a string option.

    :   **Returns:**: the new Option

---


### addIntegerOption

```
public final CmdLineParser.Option addIntegerOption(char shortForm,
                                                   java.lang.String longForm)
```

:   Convenience method for adding an integer option.

    :   **Returns:**: the new Option

---


### addIntegerOption

```
public final CmdLineParser.Option addIntegerOption(java.lang.String longForm)
```

:   Convenience method for adding an integer option.

    :   **Returns:**: the new Option

---


### addLongOption

```
public final CmdLineParser.Option addLongOption(char shortForm,
                                                java.lang.String longForm)
```

:   Convenience method for adding a long integer option.

    :   **Returns:**: the new Option

---


### addLongOption

```
public final CmdLineParser.Option addLongOption(java.lang.String longForm)
```

:   Convenience method for adding a long integer option.

    :   **Returns:**: the new Option

---


### addDoubleOption

```
public final CmdLineParser.Option addDoubleOption(char shortForm,
                                                  java.lang.String longForm)
```

:   Convenience method for adding a double option.

    :   **Returns:**: the new Option

---


### addDoubleOption

```
public final CmdLineParser.Option addDoubleOption(java.lang.String longForm)
```

:   Convenience method for adding a double option.

    :   **Returns:**: the new Option

---


### addBooleanOption

```
public final CmdLineParser.Option addBooleanOption(char shortForm,
                                                   java.lang.String longForm)
```

:   Convenience method for adding a boolean option.

    :   **Returns:**: the new Option

---


### addBooleanOption

```
public final CmdLineParser.Option addBooleanOption(java.lang.String longForm)
```

:   Convenience method for adding a boolean option.

    :   **Returns:**: the new Option

---


### getOptionValue

```
public final java.lang.Object getOptionValue(CmdLineParser.Option o)
```

:   Equivalent to `getOptionValue(o,
    null)`.

---


### getOptionValue

```
public final java.lang.Object getOptionValue(CmdLineParser.Option o,
                                             java.lang.Object def)
```

:   **Returns:**: the parsed value of the given Option, or null if the option was not set

---


### getOptionValues

```
public final java.util.Vector getOptionValues(CmdLineParser.Option option)
```

:   **Returns:**: A Vector giving the parsed values of all the occurrences of the given Option, or an empty Vector if the option was not set.

---


### getRemainingArgs

```
public final java.lang.String[] getRemainingArgs()
```

:   **Returns:**: the non-option arguments

---


### parse

```
public final void parse(java.lang.String[] argv)
                 throws CmdLineParser.IllegalOptionValueException,
                        CmdLineParser.UnknownOptionException
```

:   Extract the options and non-option arguments from the given
    list of command-line arguments. The default locale is used for
    parsing options whose values might be locale-specific.

    :   **Throws:**: `CmdLineParser.IllegalOptionValueException`: `CmdLineParser.UnknownOptionException`

---


### parse

```
public final void parse(java.lang.String[] argv,
                        java.util.Locale locale)
                 throws CmdLineParser.IllegalOptionValueException,
                        CmdLineParser.UnknownOptionException
```

:   Extract the options and non-option arguments from the given
    list of command-line arguments. The specified locale is used for
    parsing options whose values might be locale-specific.

    :   **Throws:**: `CmdLineParser.IllegalOptionValueException`: `CmdLineParser.UnknownOptionException`


---


|  |  |  |
| --- | --- | --- |
| **Overview** \| **Package** \| **Class** \| **Use** \| **Tree** \| **Deprecated** \| **Index** \| **Help** | |  |
| **PREV CLASS**   **NEXT CLASS** | **FRAMES**    **NO FRAMES**     **All Classes** |  |
| SUMMARY: NESTED \| FIELD \| CONSTR \| METHOD | DETAIL: FIELD \| CONSTR \| METHOD |  |


---
